# A risk assessment study of SARS-CoV-2 propagation in the manufacturing of cellular products

**DOI:** 10.2217/rme-2022-0096

**Published:** 2022-12-01

**Authors:** Enric Redondo Monte, David O'Neill, Karin M Abitorabi

**Affiliations:** ^1^Minaris Regenerative Medicine GmbH. Haidgraben 5, Ottobrunn, 85521, Germany; ^2^Minaris Regenerative Medicine, LLC. 4 Pearl Ct, Allendale, NJ 07401, USA

**Keywords:** ACE2, cell therapy, compliance, COVID-19, gene therapy, manufacturing, propagation, risk assessment, SARS-CoV-2, TMPRSS2

## Abstract

The potential infection of cellular therapies by SARS-CoV-2 present high risks, as the target patients for these treatments are often immunocompromised or have chronic diseases associated with a higher risk of serious illness and death by COVID-19. The multicellular tropism of this virus presents challenges for the manufacturing of cell therapies, whereby the material could potentially become infected at the source or during cell processing. In this review we assess the risk of a SARS-CoV-2 propagation in cell types used to date in cellular therapies. Altogether, the risk of SARS-CoV-2 contamination of cellular products remains low. This risk should be evaluated on an individual basis, considering ACE2 and TMPRSS2 expression, existing literature regarding the susceptibility to infection, and single cell RNA sequencing data of COVID-19 patients. This analysis should ideally be performed for both the cells being manufactured and the cells used to produce the vector to ensure patient safety.

## Plain language summary

Cell therapies are medicines based on the utilization of different cell types that are manufactured in special facilities. SARS-CoV-2, the virus that causes COVID-19, can infect a wide range of cell types. Patients requiring a cell therapy may be at higher risk of severe COVID-19 due to their underlying medical conditions. In this context, it is of importance to evaluate the risk of a SARS-CoV-2 contamination during the production of cell therapies to avoid possible infections. In this review, the authors assess the risk of an infection for cells being used as therapies to date and propose a systematic way to evaluate this risk.

## Tweetable abstract

In this review the authors explore the multicellular tropism of SARS-CoV-2 and the corresponding challenges for the manufacturing of cell therapies, where the material could potentially get infected at the source or during cell processing.

Severe acute respiratory syndrome coronavirus 2 (SARS-CoV-2) was first identified in December 2019, with subsequent rapid spread across the globe. The virus causes COVID-19, a respiratory disease that affects persons of all ages but is particularly deadly in the elderly and in patients with pre-existing conditions [1,2]. Importantly, patients who may require treatment with cell therapies are generally less fit and present a higher risk of serious illness and death due to COVID-19 [3]. For example, the adjusted risk ratio (aRR) of death from COVID-19 is 1.39 for patients who are immunosuppressed, 1.28 for patients with cardiovascular disease and 1.31 for patients with chronic lung disease of [3]. SARS-CoV-2 uses the human protein ACE2 as entry receptor through its receptor-binding domain in its spike protein [4,5]. Glycan-recognizing C-type lectins, CD209 and CD209L, which have a broad virus tropism have also been suggested as entry receptors for SARS-CoV-2 [5,6], as well as AXL, NRP1 and TIM1 [7–10]. Once internalized, the virus needs to be proteolytically activated by human proteases through a priming of its spike protein. TMPRSS2 plays a major role in this process, but other proteases such as lysosomal cathepsins may be involved in its absence [4,11]. These molecules are expressed across a variety of organs (Figure 1). ACE2, for example, is highly expressed in respiratory and intestinal epithelium [12] and in cardiac pericytes [13]. Additionally, there is direct evidence that exposure to SARS-CoV-2 produces multiorgan invasion, pointing to a multiorgan tropism of this virus [14].

**Figure 1.**
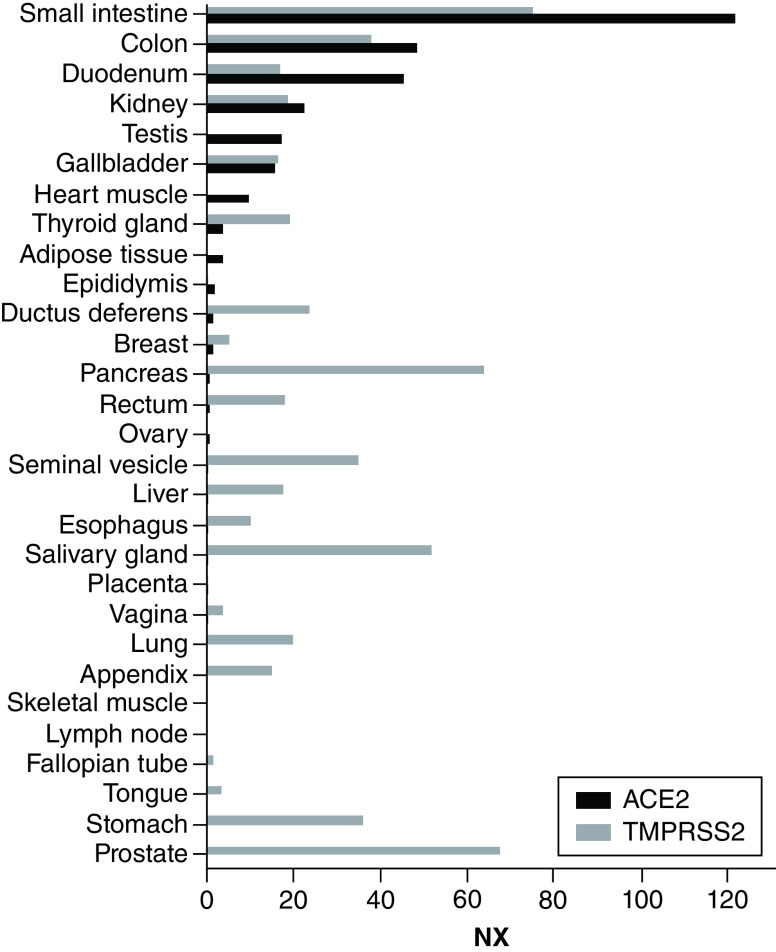
*ACE2* and *TMPRSS2* RNA expression across different human tissues. NX: Consensus Normalized eXpression from 3 transcriptomics datasets (HPA, GTEx and FANTOM5). Only tissues with an expression equal or higher than 0.5 are shown. Data available from the Human Protein Atlas version 20.1 and Ensembl version 92.38. (www.proteinatlas.org/ENSG00000130234-ACE2/tissue and www.proteinatlas.org/ENSG00000184012-TMPRSS2/tissue taken from [15]).

This broad tropism presents potential challenges for the manufacturing of cell therapies, whereby the material could conceivably become infected at the source (incoming or starting cell material) or during cell processing (final product). Cellular therapies are advanced therapeutic medicinal products (ATMPs) that are produced under current good manufacturing practices (cGMP). In USA, cGMP regulations are defined in the US Code of Federal Regulations (CFR) and in the European Union in the EudraLex Volume 4 and include, among others, strict aseptic methodology during the processing, packaging, or holding of drug products, extensive documentation, sterility testing and the path for product approval. Some ATMPs may also be manufactured under Good Tissue Practice (GTP). GTP requirements are less comprehensive than GMP requirements and are aimed primarily to avoid product contamination with transmissible agents.

Since these products contain human cells, the risk of the cells being infected by a human pathogen must be taken into consideration. Extensive rules and guidelines are in place to test the products for release. These tests include checking the cell donors for the presence of infectious disease, including the human immunodeficiency virus and hepatitis B and C viruses as defined in the CFR title 21 section 1271.75 [16]. In January 2021, the US FDA posted updated recommendations on COVID-19 regarding Human Cells, Tissues, and Cellular and Tissue-Based Products (HCT/P's) and blood establishments [17]. At the time of this update, the FDA indicated that there had been no reported cases of transmission of COVID-19 via HCT/Ps or blood products.

However unlikely, ATMPs manufacturers must prevent or mitigate potential risks on the quality and/or safety of their products. There are two main channels which could lead to the contamination of ATMPs with SARS-CoV-2. First, the donor of the cells or tissue may be infected at the time of the donation. The FDA does not currently recommend molecular testing to screen asymptomatic cell and tissue donors [17]. Nevertheless, it was recommended to take into consideration if the donor was in close contact with a COVID-19 patient, had a diagnosis of COVID-19 or a positive SARS-CoV-2 test 28 days prior to donation. The FDA also considers that vaccinated individuals should still be considered as donors and that they would not need to be screened. Second, the cells could potentially get infected during product manufacturing despite the adherence to cGMP regulations. This risk would mainly depend on the susceptibility of the target cells and vector producing cells to SARS-CoV-2 infection and propagation.

The FDA recommends that ATMP manufacturers perform a risk assessment regarding the potential transmission of SARS-CoV-2 by their products and describe possible mitigation strategies [17]. The risk assessment should consider such factors as cellular and tissue source, manufacturing processes, product testing, and number of patients to potentially be treated with the ATMP. In this review we assess the risk of SARS-CoV-2 infection for the cell types used to date in cellular therapies with approval designation (PRIME Designation, RMAT Designation, Breakthrough Designation, Fast Track Designation and SAKIGAKE Designation) in Europe, the USA and Japan [18].

## Blood-derived products

Different blood products can be used as starting material to produce cell therapies (i.e., whole blood, peripheral blood mononuclear cells, bone marrow mononuclear cell and cord blood). Broadly speaking, blood cells express low levels of *ACE2* and *TMPRSS2* [15]. However, it is important to consider that cellular types present in this kind of products may be susceptible to SARS-CoV-2.

### B cells

Single-cell RNA sequencing (scRNASeq) of COVID-19 patients has shown no evidence of B cell infection with SARS-CoV-2 [19]. Moreover, B cells express only low levels of *ACE2* and no *TMPRSS2* [15]. B cells are thus most likely not susceptible to SARS-CoV-2 infection; however, we could not find any *in vitro* studies to corroborate this affirmation. In contrast, B cell function is affected in patients who have recovered from COVID-19 [20], which may affect the functionality of cell therapies manufactured from such donors. Of importance, CD19 is also downregulated in such patients, which may have implications for the isolation of B cells through methods that relay on CD19 expression [20].

### Erythroid cells

CD34^neg^ early erythroid progenitors express high levels of *ACE2* and *TMPRSS2*. This expression is downregulated during the course of erythroid maturation. Most interestingly, CD34^neg^ erythroid progenitors are targeted by SARS-CoV-2 *in vitro*, leading to their expansion [21,22]. These observations explain the anemia observed in COVID-19 patients, which plays a major role on the hypoxia suffered by these patients [21,22]. It is unclear whether mature erythrocytes can be infected, but most likely they could not support virus replication [22].

### Hematopoietic stem & progenitor cells

CD34^+^ hematopoietic stem and progenitor cells (HSPCs) are one of the main starting materials for cellular therapies [23]. They express low levels of *ACE2* and no *TMPRSS2* [24]. In addition, direct evidence shows that they cannot be infected by SARS-CoV-2 [21]. Of interest, CD34^+^ cell exposure to the SARS-CoV-2 Spike (S) protein alters their growth *in vitro* [24]. Cell therapies using HSPCs have been approved under the commercial names of Libmeldy^®^, Skysona^®^, Strimvelis^®^, Zolgensma^®^ and Zynteglo^®^, for the treatment of metachromatic leukodystrophy, early cerebral adrenoleukodystrophy, adenosine deaminase severe combined immune deficiency and beta thalassemia, respectively. The presence of a gene modification manufacturing step in all these therapies should be considered in the risk assessment (see vector-producing cell lines section below). All these therapies are autologous, and thus would not have a risk of SARS-CoV-2 expansion to multiple patients.

### Monocytes, macrophages & dendritic cells

The coronaviruses Middle East respiratory syndrome coronavirus (MERS-CoV) and SARS-CoV-1 are both known to infect macrophages [25,26]. Similarly, SARS-CoV-2 can infect both monocytes and macrophages, but this infection is abortive, meaning, SARS-CoV-2 virus does not replicate in these cells [27]. Infection and viral protein production without virus replication were also observed in monocyte-derived dendritic cells [28]. scRNAseq of bronchoalveolar lavage fluid from intubated COVID-19 patients detected SARS-CoV-2 sequences in monocyte-derived alveolar macrophages and migratory dendritic cells, although neither *ACE2* nor *TMPRSS2* could be detected in either cell type [15,19]. These data put into question the validity of an infection risk assessment exclusively based on the expression of the two markers mentioned above. A possible explanation to this observation could be that macrophages become infected *in vivo* after phagocytosis of infected cells in the alveoli [19]. These observations may also be explained by direct infection thorough NRP1 and AXL in dendritic cells and monocytes, or CD209 on monocytes [15]. Finally, SARS-CoV-2 could also infect these cells by means of antibody-dependent enhancement [29]. Provenge^®^ is an approved dendritic cell-based therapy against hormone refractory prostate cancer. The manufacturing of this therapy does not involve the transduction of the cells and is autologous, minimizing the risks associated with a SARS-CoV-2 infection to one patient.

### Natural killer cells

Natural killer (NK) cells do not express *ACE2* nor *TMPRSS2* [15]. scRNAseq showed no evidence of infected NK cells in the lungs of COVID-19 patients [19]. Unfortunately, to the best of our knowledge, no *in vitro* studies have been performed to date regarding the susceptibility of infection of these cells. However, data show that NK cells in COVID-19 patients present a dysfunctional status, with a decrease of numbers of circulating cells that might derive from virus-induced apoptosis [30]. In this context, testing of the donors would be recommended to avoid the manufacturing of dysfunctional NK products.

### Platelets & megakaryocytes

Platelets and megakaryocytes do not express ACE2 [31]. However, platelets can present SARS-CoV-2, as demonstrated by samples of circulating platelets of COVID-19 patients [32]. The origin of these cells may be infected megakaryocytes that differentiate into platelets, as megakaryocytes have been shown to be infected in samples from COVID-19 autopsies [32]. Other molecules like NPR1 have been proposed as a possible point of entry for SARS-CoV-2 in megakaryocytes [31]. What is more, patients with COVID-19 show a platelet hyperactivation [33]. This does not seem to be mediated by the virus itself or their proteins, but rather by the expression of different factors by the infected cells [33]. Aurix™ is an autologous platelet-based therapy for the treatment of chronic non-healing wounds. Since platelet activity may be influenced by the presence of COVID-19, a test of the donors would be recommended.

### T cells

T cells show no signs of *ACE2* nor *TMPRSS2* expression [15]. In a study, scRNAseq of COVID-19 patients showed no evidence of T cell infection [19]. Taking these two facts into account, the likelihood that T cells could be infected by SARS-CoV-2 is relatively limited, however, no studies directly testing the susceptibility of this cell type were found. Therapies using T cells as a starting cell material have been approved under the commercial names of Abecma^®^, Breyanzi^®^, Kymriah^®^, Tecartus^®^, Yescarta^®^ and Zalmoxis^®^ for treatment of relapsed or refractory multiple myeloma, relapsed or refractory large B-cell lymphoma, acute lymphoblastic leukemia, chronic lymphoid leukemia and diffuse large B-cell lymphoma, mantle cell lymphoma, non-Hodgkin lymphoma and follicular lymphoma. All these therapies include a gene modification step, which should be considered during the SARS-CoV-2 propagation risk assessment. The immune compromised state of these patients presents an additional risk for their health with relation to a SARS-CoV-2 infection.

## Non-blood derived products

### Aortic endothelial cells

A study using immunofluorescence detected both ACE2 and SARS-CoV-2 in the vascular endothelial cells of multiple organs of COVID-19 patients, although aortic endothelial cells were not examined [14]. Additionally, the presence of the virus in vascular endothelial cells was confirmed in a study using material from autopsies from COVID-19 patients, without including aortic tissue [34]. *In vitro* studies demonstrated that SARS-CoV-2 can infect and replicate in human blood vessel organoids [35]. Finally, *in vitro* studies also showed that the presence of SARS-CoV-2 nucleocapsid proteins activated and damaged aortic endothelial cells, offering a possible explanation for the endothelial injury observed in some COVID-19 patients [36].

### Cardiosphere-derived cells

Cardiosphere-derived cells (CDCs) are a progenitor cell population found in the heart [37]. Co-expression of ACE2 and the spike protein of SARS-CoV-2 has been observed in the heart of COVID-19 patients [14]. Moreover, the Human Protein Atlas shows that all the tested cell types present in the heart express *ACE2* and low levels of *TMPRSS2* [15]. *In vitro* infectivity was also demonstrated in cardiomyocytes derived from induced Pluripotent Stem Cells (iPSCs) [38], cardiosphere-derived stromal cells [39] and in a cardiosphere model [40]. Moreover, infected cells can develop into a hyper-inflammatory phenotype that may explain the cardiac complications observed in COVID-19 patients [39]. Finally, patients with cardiovascular disease have a COVID-19 mortality aRR of 1.28, highlighting the importance of preventing SARS-CoV-2 infection in patients who need cardiac-derived cell therapies [3].

### Human embryonic stem cells

Human embryonic stem cells (hESCs) express low levels of both *ACE2* and *TMPRSS2* [41]. No information could be found regarding the susceptibility of hESCs to SARS-CoV-2 infection, but the fact that iPSCs are refractory to the infection may point to a similar phenotype for hESCs [42]. Importantly, cells differentiated from hESCs can become susceptible to the infection, especially if the daughter cells express high levels of ACE2 and TMPRSS2 [43–45].

### Induced pluripotent stem cells

iPSCs express low levels of both *ACE2* and *TMPRSS2* [41] and are refractory to SARS-CoV-2 infection [42]. Nonetheless, it should be considered that iPSCs may present different expression profiles and differentiation capacities depending on their tissue of origin and donor characteristics which could theoretically influence SARS-CoV-2 susceptibility [46,47]. As in the case of hESC, more differentiated cells derived from iPSCs can become susceptible to the infection, especially if the daughter cells express high levels of ACE2 and TMPRSS2. As an example, derived lung, neural and cardiac cells are permissive for SARS-CoV-2 infection *in vitro* [48,49].

### Mesenchymal stem & progenitor cells

Mesenchymal stem cells (MSCs) do not express ACE2 nor TMPRSS2, and thus would not be expected to be targets for SARS-CoV-2 infection [50]. In addition, there are published reports that MSCs are resistant to SARS-CoV-2 infection *in vitro* [50,51]. These data are especially encouraging due to the potential use of MSCs as treatment for COVID-19 [52,53]. Alofisel^®^, Stemirac^®^ and Temcell^®^, all MSC-based products, have gained market authorization for the treatment of, among others, chronic obstructive pulmonary disease, graft-versus-host disease, Type I diabetes, and myocardial infarction: all diseases with an increased risk of death by COVID-19 [3]. These are all allogenic therapies, increasing the importance of a proper risk assessment.

### Oligodendrocyte progenitor cells

Oligodendrocyte progenitor cells (OPCs) express both *ACE2* and *TMPRSS2* [54], making them potential targets for SARS-CoV-2. A study using BrainSpheres (organoids derived from iPSCs containing neurons, astrocytes, and oligodendrocytes) demonstrated that SARS-CoV-2 can infect and replicate in this system, but a definitive statement of which cell types were infected was not made [55]. Recently a report showed that OPCs overexpress genes related to COVID-19 pathogenesis which suggests that they may be susceptible to infection [56].

### Discogenic cells

Discogenic cells are progenitor cells originating from adult human intervertebral disc tissue, specifically from the nucleus pulposus tissue, and have a notochordal origin [57]. These cells exhibit a multipotency for mesenchymal lineage differentiation [58] which could suggest a similar phenotype to MSCs, namely no ACE2 and TMPRSS2 expression and no susceptibility to coronavirus infection [50,51]. However, no literature could be found regarding these topics for this cell type.

### Retinal cells

An *in vitro* model based on hESC-derived eye organoids demonstrated that retinal pigment epithelial (RPE) cells express ACE2 and TMPRSS2 and are targeted by SARS-CoV-2 [59]. Moreover, iPSC-derived retinal organoids express ACE2 and TMPRSS2 and a SARS-CoV-2 spike protein pseudovirus can enter these cells [60]. These findings are consistent with the fact that COVID-19 patients present retinal manifestations of the disease [61]. No cell therapies using retinal cells have been approved so far, but Luxturna^®^, an AVV based gene therapy, has been approved for the treatment of Leber congenital amaurosis.

### Vector producing cell lines

Viral vectors (often based on retroviruses and lentiviruses) are used to transduce cells to express specific proteins in ATMPs. Commonly, human embryonic kidney-derived cell lines (HEK293 and HEK 293T cells) are used for viral vector production [62,63], but these cells express only low levels of ACE2 [15]. These lines have been shown to be susceptible to SARS-CoV-2 infection, but only with a limited viral replication capacity [64]. Interestingly, HEK293 and 293T cells transfected with *ACE2* and *TMPRSS2* can be easily infected by SARS-CoV-2 [11,65].

The human cervical adenocarcinoma cell line HeLa has also been used for viral vector packaging [62]. HeLa cells do not express ACE2 [66] and are not naturally infected by the SARS-CoV-2 [67]. However, HeLa cells transfected with an *ACE2* plasmid (transiently expressing ACE2) are capable of being infected [68].

The Vero line is a monkey kidney epithelial cell line which expresses ACE2 [69] and can be infected by the SARS-CoV-2 virus [69–71]. Although our literature search found no evidence that Vero cells are used for GMP production of gene therapy viral vectors, they have been used in other manufacturing processes such as in vaccine production [72].

## Discussion

The COVID-19 pandemic caused by the SARS-CoV-2 virus has forced many industries to adapt their way of working, having also had a great impact on the ATMP field [73]. As shown in this article, different cell types present highly distinct susceptibilities to SARS-CoV-2 infection. These range from cells which cannot be infected by the virus (i.e., MSCs), to cells completely receptive to virus infection (i.e., endothelial cells), including cells which can be infected but show no virus replication (i.e., dendritic cells). A summary of the susceptibility to SARS-CoV-2 infection of the cellular types discussed in this review is shown in Table 1. A summary of the therapies with full approval in Japan, the EU and the USA and their cellular material is shown in Table 2.

**Table 1.** Summary of the risk assessment of SARS-CoV-2 infection for all the cell types used to date in cellular therapies with approval designation (PRIME Designation, RMAT Designation, Breakthrough Designation, Fast Track Designation and SAKIGAKE Designation) in Europe, the USA and Japan.

Material	*ACE2* expression	*TMPRSS2* expression	Infection susceptibility	Additional infection information	Other considerations	Refs.
Aortic endothelial cells	Yes	No	Yes	Infection and replication in human blood vessel organoids	Activated by SARS-CoV-2 nucleocapsid protein	[14,35,36]
B cells	Low	No	No	scRNASeq no evidence of B cell infection		[15,19]
Blood products (PBMC, whole blood, bone marrow)	Expression in some cell types	No	Yes	Some small subsets of cells are susceptible	Susceptible cell types may not part of the final cell product	[15,19,21]
Cardiac progenitor cells	Yes	Low	Yes		Infected cells can cause a hyper-inflammation	[14,38-40]
Dendritic cells	Low	Yes	Yes	Permissive to infection and protein expression but not virus replication		[28]
Erythroid cells	Yes (early erythroid progenitors)	Yes (early erythroid progenitors)	Yes	CD34- early erythroid progenitors susceptible to SARS-CoV-2 infection with viral replication	Cause of anemia in COVID-19 patients	[21]
HEK293T (embryonic kidney)	No	No	Yes	Susceptible infection, low viral replication	Transfection with ACE2 and TMPRSS2 allows infection	[15,64,65]
HeLa (cervical cancer)	No	No	No		Transfection with an ACE2 plasmid allows for infection	[15,66-68]
Hematopoietic stem and progenitor cells	Low	No	No		Exposure to S protein alters proliferation/expansion	[21,24]
Human embryonic stem cells	Low	Low	No information available			[41]
Induced pluripotent stem cells	Low	Low	No		Derived lung, neural and cardiac cells permissive to infection	[41,42,48,49]
Mesenchymal stem cells	No	No	No			[50,51,53]
Monocytes and macrophages	No	No	Yes	Susceptible, but the infection is abortive		[15,27]
Natural killer cells	No	No	No information available			[15]
Oligodendrocyte progenitor cells	Yes	Yes	No information available		Overexpression of genes related to COVID-19	[54-56]
Progenitor cells from intervertebral disc tissue	No information available	No information available	No information available			
Retinal cells	Yes	Yes	Yes		COVID-19 patients have retinal symptoms	[59-61]
T cells	No	No	No	scRNASeq no evidence of infection		[15,19]
Vero (Monkey kidney epithelial)	Yes	No	Yes			[69–71]

PBMC: Peripheral blood mononuclear cell; scRNASeq: Single cell RNA sequencing.

**Table 2.** Summary of therapeutic cell types with products with full approval in either Japan, the EU or the USA.

Material	Indications	Commercial products
Blood products (PBMC, whole blood, bone marrow)	Treatment of wounds	Aurix™
Dendritic cells	Hormone refractory prostate cancer	Provenge^®^
Hematopoietic stem and progenitor cells	Metachromatic leukodystrophy, early Cerebral Adrenoleukodystrophy, adenosine deaminase severe combined immune deficiency, beta thalassemia	Libmeldy^®^, Skysona^®^, Strimvelis^®^, Zolgensma^®^, Zynteglo^®^
Mesenchymal stem cells	Crohn's disease, spinal cord injury, acute radiation injury, chronic obstructive pulmonary disease, graft-vs-host disease, Type I diabetes and myocardial infarction	Alofisel^®^, Stemirac^®^, Temcell^®^
Retinal cells (as target cell)	Leber congenital amaurosis	Luxturna^®^
T cells	Relapsed or refractory multiple myeloma, relapsed or refractory large B-cell lymphoma, acute lymphoblastic leukemia, chronic lymphoid leukemia and diffuse large B-cell lymphoma, mantle cell lymphoma, non-Hodgkin lymphoma, follicular lymphoma	Abecma^®^, Breyanzi^®^, Kymriah^®^, Tecartus^®^, Yescarta^®^, Zalmoxis^®^

PBMC: Peripheral blood mononuclear cell.

An ATMP could potentially become infected at the source (incoming or starting cell material) or during cell processing (final product). This was the case during the Zika virus outbreak, which could be transmitted through blood products and semen [74,75]. This fact led the FDA to recommend persons diagnosed with Zika virus infection in the previous 6 months or that visited a Zika high-prevalence area to be ineligible as cell and tissue donors [16]. To date, there have not yet been cases of transmission of COVID-19 via HCT/Ps or blood products reported in the literature. The FDA also states that it is not aware of any case of transmission via these products [17]. Additionally, coronaviruses are not known to be transmitted by HCT/P transplants [17]. However, it is the responsibility of the manufacturers to prevent this risk. It may be beneficial from a risk management perspective to test cell donors for SARS-CoV-2 if they have been in close contact with a known positive, have had symptoms, have had a positive SARS-CoV-2 test, or have developed COVID-19 in the last 28 days, as recommended by the FDA [17]. The risk of SARS-CoV-2 transmission from an ATMP into a patient would be highest for allogeneic cell lines/banks of epithelial or endothelial cells that express ACE2 and TMPRSS2. Such cell banks could be used for multiple (patient non-specific) doses of final product, so safety measures and potentially donor screening should be implemented.

During cell processing an ATMP could potentially become contaminated in two ways. First, the product may be contaminated due to direct contact with an operator suffering COVID-19. Considering the strict sterility and hygiene standards held by the industry, a direct contamination of the cell product from an operator is highly unlikely. However, testing, mask mandates and vaccination are still beneficial to prevent virus spread between operators and would minimize the risk of contamination of the product. A second way a product could potentially be contaminated would be through cell culture materials of human origin used during the manufacturing process, which could have been contaminated at the source. Currently, the FDA does not recommend testing incoming materials for SARS-CoV-2 [17]. The risk would be unlikely for products such as albumin USP, which has alcohol fractionation and is pasteurized (60°C for 10 h) to inactivate viruses (e.g., see CSL Behring Albuminar-5 factsheet). The risk could, however, be higher for pooled human serum, which does not use these types of processes. For pooled serum, donors are screened and tested following FDA guidelines, which do not currently include testing for SARS-CoV-2 [17]. A close collaboration with suppliers of biological materials of human origin to make sure they are taking all adequate measures to avoid contamination of such products will be necessary. It is important to remark that the current cleaning procedures used in cleanrooms for ATMP production relay on high-level disinfectants, based on isopropanol, bleach, hydrogen peroxide and phenols. These disinfectants are adequate for the destruction of coronaviruses, further minimizing the risk of a product contamination [76].

Although a seemingly good strategy to avoid a SARS-CoV-2 infection of an ATMP would be to test the operators, donors, and the cell product all the way through the production of the drug product, several challenges make this approach very difficult to implement. First, the availability of the material for testing is limited, with many processes relaying on precious material where every cell counts toward the success of the therapy. Second, there is a financial consideration, with serial SARS-CoV-2 testing adding to the financial burden of producing ATMPs. Finally, the willingness of the operators and donors to get tested may also suppose a limitation to this broad testing strategy.

Importantly, a risk assessment should ideally be based on experimental data and not only on annotations of ACE2 and TMPRSS2 expression from existing databases. While such information may be useful, some cell types, (i.e., monocytes and dendritic cells), do not show high expression of these markers but are still susceptible to SARS-CoV-2 infection [19,27]. This fact could be explained by the role of further surface molecules and proteases in SARS-CoV-2 cell infection [5–10], limiting the value of ACE2 and TMPRSS2 as standalone markers. What is more, when using databases that assess expression at the organ level, some organs which are highly damaged in COVID-19 patients may appear to have a low expression of these markers (i.e., the lung, Figure 1). This may be an effect of the low resolution of this kind of data, for example, only epithelial cells may be susceptible to infection, but the lung is a heterogeneous tissue formed by several types of cells. Data with a cellular resolution is always to be preferred when performing a risk assessment for an ATMP.

## Conclusion

**Figure 2.**
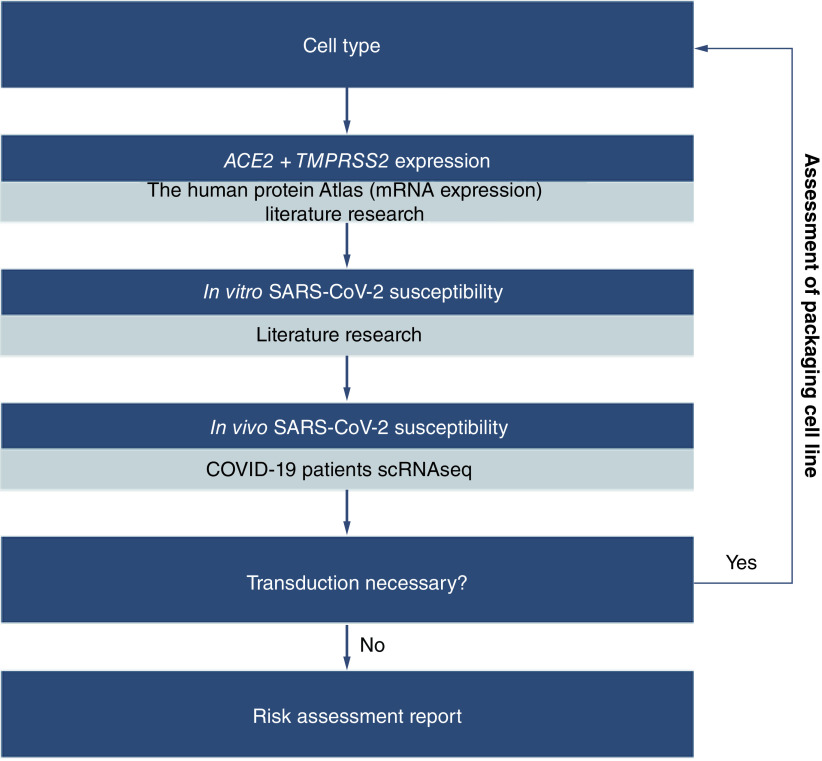
Strategy for the risk assessment of SARS-CoV-2 propagation in cell types used for the manufacturing of advanced therapies. scRNAseq: Single-cell RNA sequencing.

Altogether, the risk of SARS-CoV-2 contamination of cellular products is low but not non-existent. The risk should be evaluated on an individual basis for each therapy being produced. The expression of ACE2 and TMPRSS2 should be investigated for the cell type being used. A literature review on *in vitro* experiments evaluating the susceptibility to virus infection and primary scRNASeq data of COVID-19 patients should complete this risk evaluation (Figure 2). This evaluation should be performed for both the cells being manufactured as well as the vector-producing cells.

## Future perspective

As pandemics become more frequent due to factors such as overpopulation, globalization (e.g., easy travel), and climate change [77], stakeholders in the development and manufacturing of cell therapies will need to keep their processes and analytical methods up to date to comply with an ever-evolving regulatory environment. However, the tendency of the industry toward automation and the development of single-use closed systems will mitigate the risks of propagation of these pathogens during manufacturing. Moreover, technological advances in diagnostic methods tend to ever cheaper, faster, and reliable tests that will allow to screen cell and other human materials for use in cell therapy manufacturing without significantly increasing costs and time of production.

**Executive summary**

- SARS-CoV-2 presents a multicellular tropism.
- Patients requiring cell therapies have a higher risk of serious illness and death due to COVID-19.
- The risk of SARS-CoV-2 propagation in manufacturing needs to be evaluated and minimized.
- Cell material could potentially be infected through the donor or could get contaminated during cell manufacturing.
- Each cell type presents different susceptibility to SARS-CoV-2 infection.
- Cell tropism can be evaluated through *ACE2* and *TMPRSS2* RNA expression, literature regarding *in vitro* infectability, and single-cell RNA sequencing data of COVID-19 patients.
- These types of analysis should be performed for both the therapeutic cells being manufactured and, in the case of gene therapies, the cells used to produce the vector.
- Similarly, each therapy and manufacturing process present its own specific risks.
- Strict sterility standards (personal and environmental monitoring) held by the manufacturers make a direct contamination from an operator highly unlikely.
- Cell culture materials of human origin could be contaminated at the source and may need to be tested.
- The risk should be evaluated on an individual basis for each therapy being produced, with the publication providing valuable information for this assessment.
